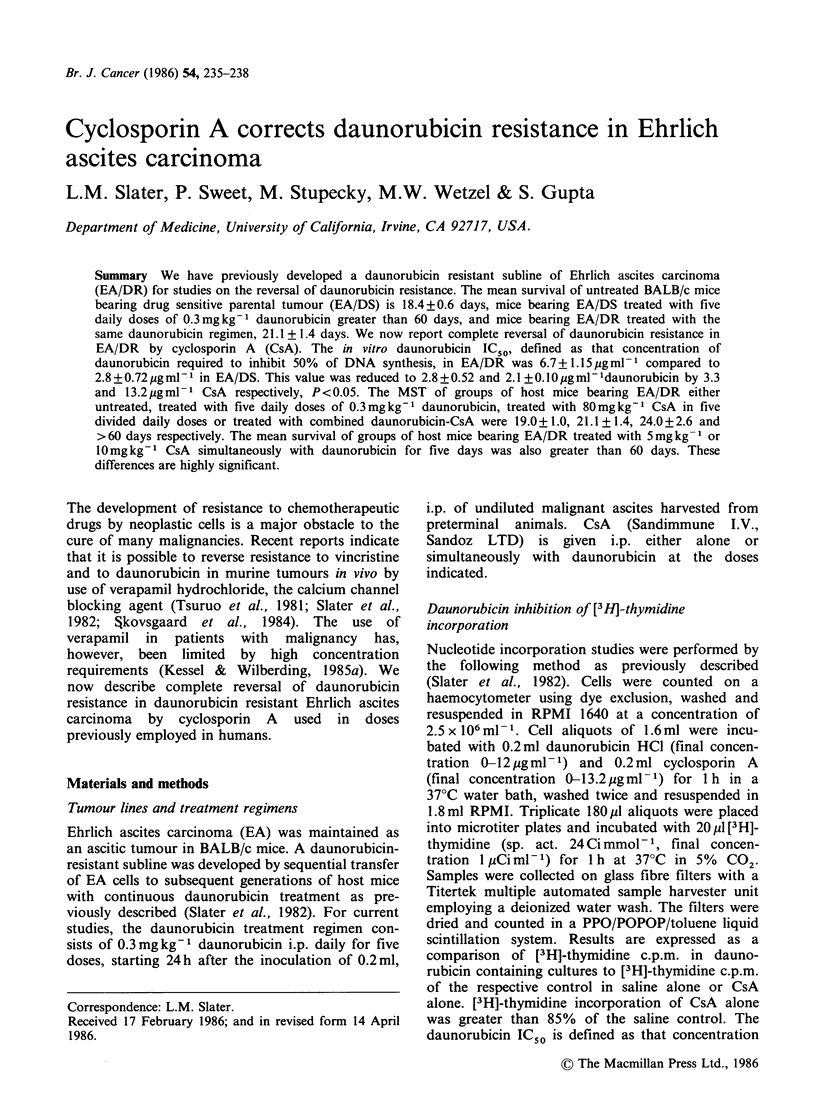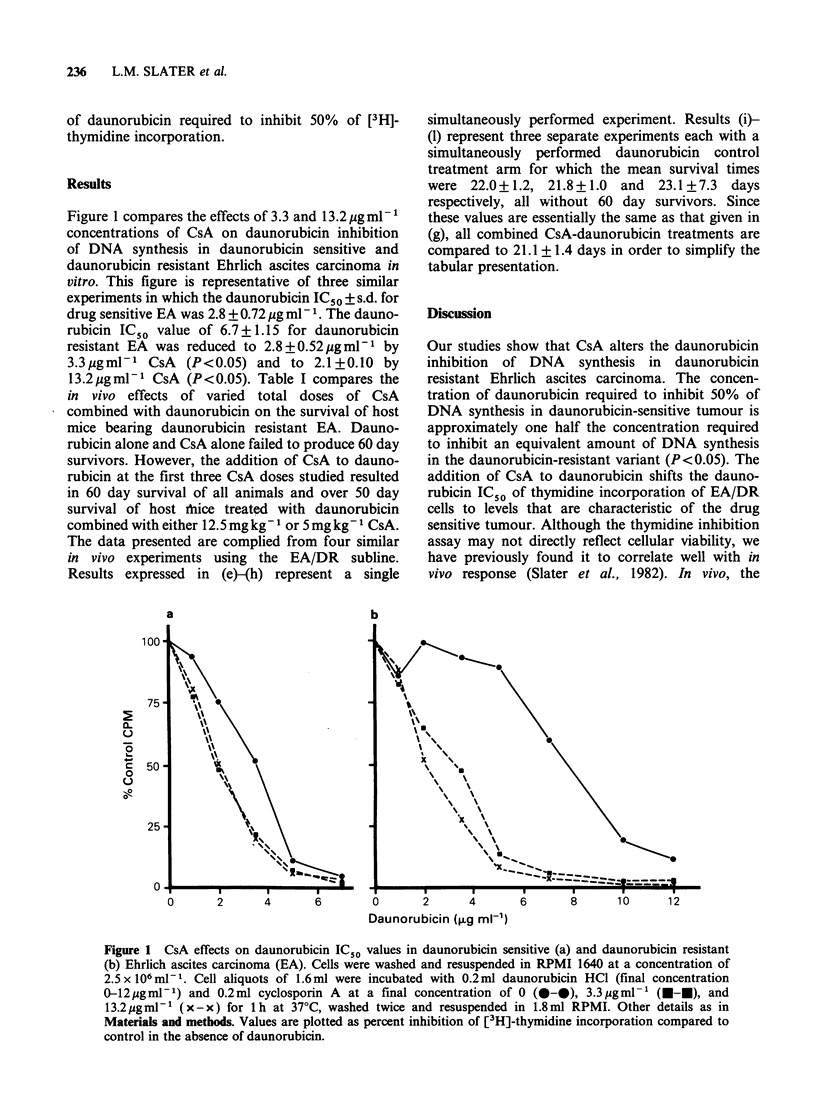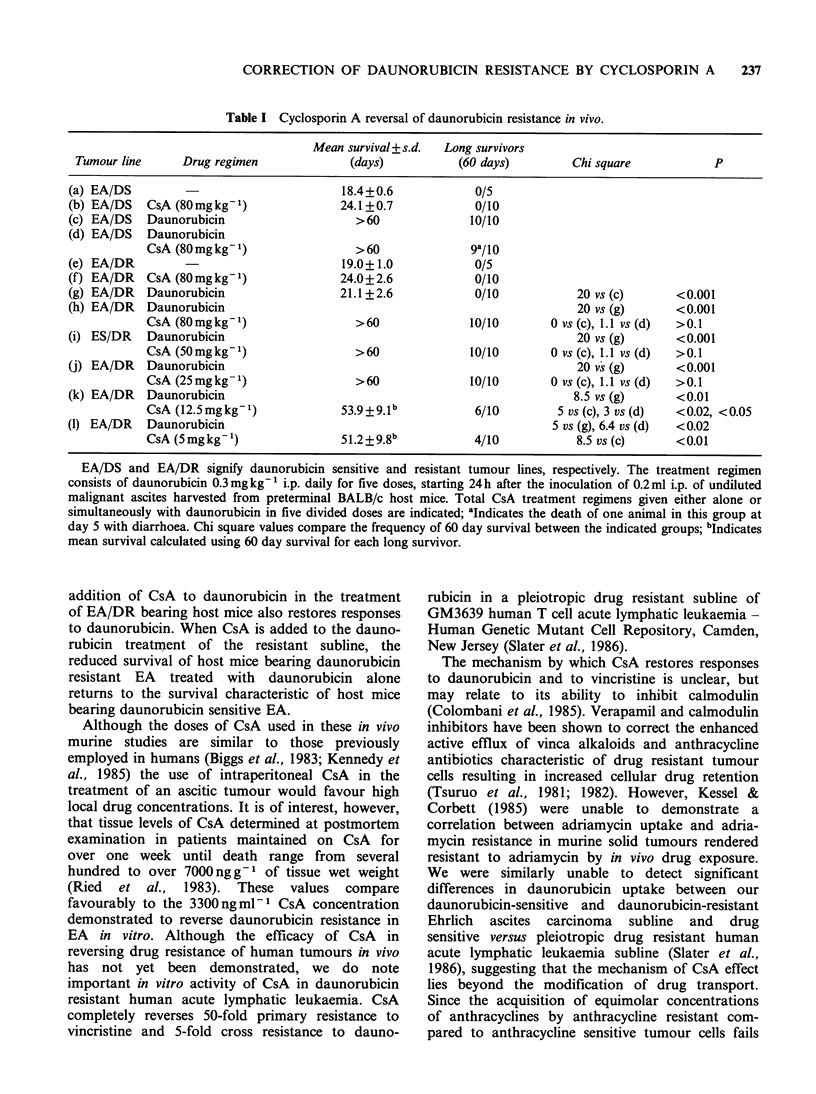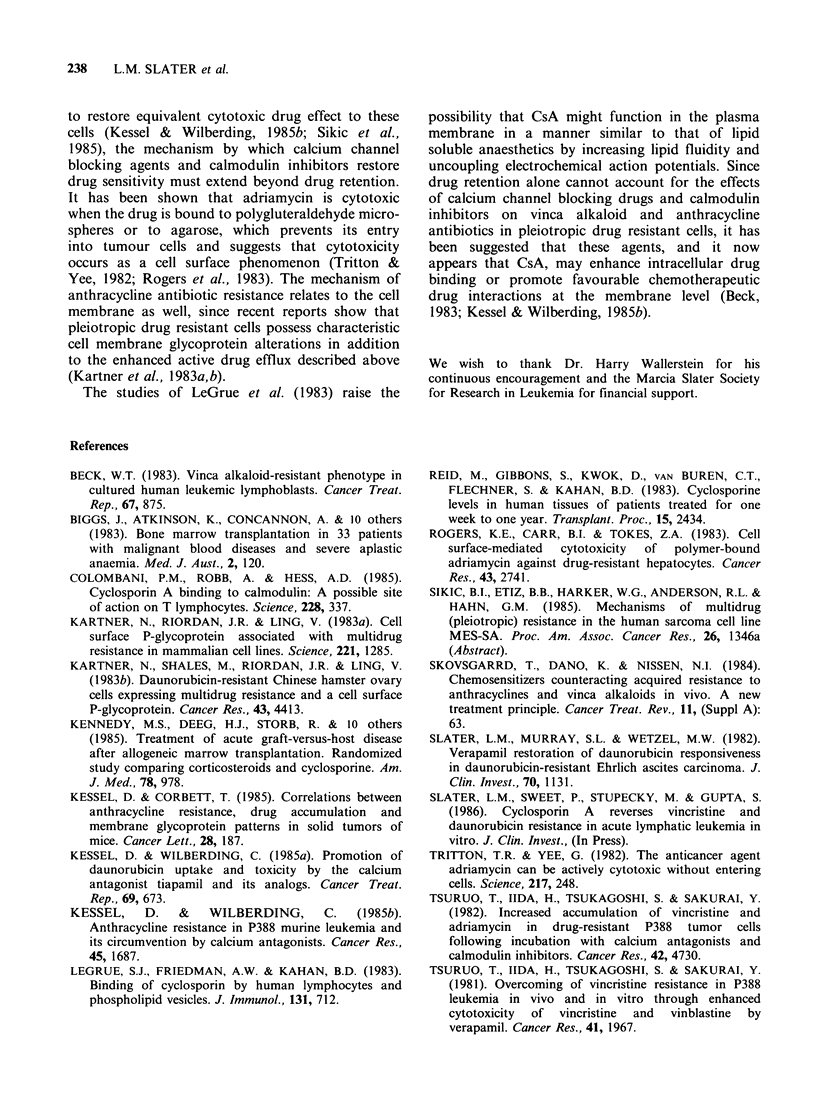# Cyclosporin A corrects daunorubicin resistance in Ehrlich ascites carcinoma.

**DOI:** 10.1038/bjc.1986.167

**Published:** 1986-08

**Authors:** L. M. Slater, P. Sweet, M. Stupecky, M. W. Wetzel, S. Gupta

## Abstract

We have previously developed a daunorubicin resistant subline of Ehrlich ascites carcinoma (EA/DR) for studies on the reversal of daunorubicin resistance. The mean survival of untreated BALB/c mice bearing drug sensitive parental tumour (EA/DS) is 18.4 +/- 0.6 days, mice bearing EA/DS treated with five daily doses of 0.3 mg kg-1 daunorubicin greater than 60 days, and mice bearing EA/DR treated with the same daunorubicin regimen, 21.1 +/- 1.4 days. We now report complete reversal of daunorubicin resistance in EA/DR by cyclosporin A (CsA). The in vitro daunorubicin IC50, defined as that concentration of daunorubicin required to inhibit 50% of DNA synthesis, in EA/DR was 6.7 +/- 1.15 micrograms ml-1 compared to 2.8 +/- 0.72 micrograms ml-1 in EA/DS. This value was reduced to 2.8 +/- 0.52 and 2.1 +/- 0.10 micrograms ml-1 daunorubicin by 3.3 and 13.2 micrograms ml-1 CsA respectively, P less than 0.05. The MST of groups of host mice bearing EA/DR either untreated, treated with five daily doses of 0.3 mg kg-1 daunorubicin, treated with 80 mg kg-1 CsA in five divided daily doses or treated with combined daunorubicin-CsA were 19.0 +/- 1.0, 21.1 +/- 1.4, 24.0 +/- 2.6 and greater than 60 days respectively. The mean survival of groups of host mice bearing EA/DR treated with 5 mg kg-1 or 10 mg kg-1 CsA simultaneously with daunorubicin for five days was also greater than 60 days. These differences are highly significant.


*Br. J. Cancer* (1986) **54**, 235–238

### Cyclosporin A corrects daunorubicin resistance in Ehrlich ascites carcinoma

L.M. Slater, P. Sweet, M. Stupecky, M.W. Wetzel & S. Gupta

*Department of Medicine, University of California, Irvine, CA 92717, USA.*

**Summary** We have previously developed a daunorubicin resistant subline of Ehrlich ascites carcinoma (EA/DR) for studies on the reversal of daunorubicin resistance. The mean survival of untreated BALB/c mice bearing drug sensitive parental tumour (EA/DS) is $18.4 \pm 0.6$ days, mice bearing EA/DS treated with five daily doses of $0.3\,\mathrm{mg\,kg^{-1}}$ daunorubicin greater than 60 days, and mice bearing EA/DR treated with the same daunorubicin regimen, $21.1 \pm 1.4$ days. We now report complete reversal of daunorubicin resistance in EA/DR by cyclosporin A (CsA). The *in vitro* daunorubicin $IC_{50}$, defined as that concentration of daunorubicin required to inhibit 50% of DNA synthesis, in EA/DR was $6.7 \pm 1.15\,\mu\mathrm{g\,ml^{-1}}$ compared to $2.8 \pm 0.72\,\mu\mathrm{g\,ml^{-1}}$ in EA/DS. This value was reduced to $2.8 \pm 0.52$ and $2.1 \pm 0.10\,\mu\mathrm{g\,ml^{-1}}$daunorubicin by 3.3 and $13.2\,\mu\mathrm{g\,ml^{-1}}$ CsA respectively, $P<0.05$. The MST of groups of host mice bearing EA/DR either untreated, treated with five daily doses of $0.3\,\mathrm{mg\,kg^{-1}}$ daunorubicin, treated with $80\,\mathrm{mg\,kg^{-1}}$ CsA in five divided daily doses or treated with combined daunorubicin-CsA were $19.0 \pm 1.0$, $21.1 \pm 1.4$, $24.0 \pm 2.6$ and $>60$ days respectively. The mean survival of groups of host mice bearing EA/DR treated with $5\,\mathrm{mg\,kg^{-1}}$ or $10\,\mathrm{mg\,kg^{-1}}$ CsA simultaneously with daunorubicin for five days was also greater than 60 days. These differences are highly significant.

The development of resistance to chemotherapeutic drugs by neoplastic cells is a major obstacle to the cure of many malignancies. Recent reports indicate that it is possible to reverse resistance to vincristine and to daunorubicin in murine tumours *in vivo* by use of verapamil hydrochloride, the calcium channel blocking agent (Tsuruo *et al.*, 1981; Slater *et al.*, 1982; Şkovsgaard *et al.*, 1984). The use of verapamil in patients with malignancy has, however, been limited by high concentration requirements (Kessel & Wilberding, 1985*a*). We now describe complete reversal of daunorubicin resistance in daunorubicin resistant Ehrlich ascites carcinoma by cyclosporin A used in doses previously employed in humans.

### Materials and methods

### *Tumour lines and treatment regimens*

Ehrlich ascites carcinoma (EA) was maintained as an ascitic tumour in BALB/c mice. A daunorubicin-resistant subline was developed by sequential transfer of EA cells to subsequent generations of host mice with continuous daunorubicin treatment as previously described (Slater *et al.*, 1982). For current studies, the daunorubicin treatment regimen consists of $0.3\,\mathrm{mg\,kg^{-1}}$ daunorubicin i.p. daily for five doses, starting 24 h after the inoculation of 0.2 ml, i.p. of undiluted malignant ascites harvested from preterminal animals. CsA (Sandimmune I.V., Sandoz LTD) is given i.p. either alone or simultaneously with daunorubicin at the doses indicated.

### *Daunorubicin inhibition of [$^3H$]-thymidine incorporation*

Nucleotide incorporation studies were performed by the following method as previously described (Slater *et al.*, 1982). Cells were counted on a haemocytometer using dye exclusion, washed and resuspended in RPMI 1640 at a concentration of $2.5 \times 10^6\,\mathrm{ml^{-1}}$. Cell aliquots of 1.6 ml were incubated with 0.2 ml daunorubicin HCl (final concentration $0$–$12\,\mu\mathrm{g\,ml^{-1}}$) and 0.2 ml cyclosporin A (final concentration $0$–$13.2\,\mu\mathrm{g\,ml^{-1}}$) for 1 h in a 37°C water bath, washed twice and resuspended in 1.8 ml RPMI. Triplicate $180\,\mu$l aliquots were placed into microtiter plates and incubated with $20\,\mu$l [$^3$H]-thymidine (sp. act. $24\,\mathrm{Ci\,mmol^{-1}}$, final concentration $1\,\mu\mathrm{Ci\,ml^{-1}}$) for 1 h at 37°C in 5% $CO_2$. Samples were collected on glass fibre filters with a Titertek multiple automated sample harvester unit employing a deionized water wash. The filters were dried and counted in a PPO/POPOP/toluene liquid scintillation system. Results are expressed as a comparison of [$^3$H]-thymidine c.p.m. in daunorubicin containing cultures to [$^3$H]-thymidine c.p.m. of the respective control in saline alone or CsA alone. [$^3$H]-thymidine incorporation of CsA alone was greater than 85% of the saline control. The daunorubicin $IC_{50}$ is defined as that concentration

Correspondence: L.M. Slater.
Received 17 February 1986; and in revised form 14 April 1986.

© The Macmillan Press Ltd., 1986

of daunorubicin required to inhibit 50% of [$^3$H]-thymidine incorporation.

### Results

Figure 1 compares the effects of 3.3 and 13.2 $\mu$g ml$^{-1}$ concentrations of CsA on daunorubicin inhibition of DNA synthesis in daunorubicin sensitive and daunorubicin resistant Ehrlich ascites carcinoma *in vitro*. This figure is representative of three similar experiments in which the daunorubicin $IC_{50} \pm$ s.d. for drug sensitive EA was $2.8 \pm 0.72$ $\mu$g ml$^{-1}$. The daunorubicin $IC_{50}$ value of $6.7 \pm 1.15$ for daunorubicin resistant EA was reduced to $2.8 \pm 0.52$ $\mu$g ml$^{-1}$ by 3.3 $\mu$g ml$^{-1}$ CsA ($P<0.05$) and to $2.1 \pm 0.10$ by 13.2 $\mu$g ml$^{-1}$ CsA ($P<0.05$). Table I compares the *in vivo* effects of varied total doses of CsA combined with daunorubicin on the survival of host mice bearing daunorubicin resistant EA. Daunorubicin alone and CsA alone failed to produce 60 day survivors. However, the addition of CsA to daunorubicin at the first three CsA doses studied resulted in 60 day survival of all animals and over 50 day survival of host mice treated with daunorubicin combined with either 12.5 mg kg$^{-1}$ or 5 mg kg$^{-1}$ CsA. The data presented are complied from four similar *in vivo* experiments using the EA/DR subline. Results expressed in (e)–(h) represent a single simultaneously performed experiment. Results (i)–(l) represent three separate experiments each with a simultaneously performed daunorubicin control treatment arm for which the mean survival times were $22.0 \pm 1.2$, $21.8 \pm 1.0$ and $23.1 \pm 7.3$ days respectively, all without 60 day survivors. Since these values are essentially the same as that given in (g), all combined CsA-daunorubicin treatments are compared to $21.1 \pm 1.4$ days in order to simplify the tabular presentation.

### Discussion

Our studies show that CsA alters the daunorubicin inhibition of DNA synthesis in daunorubicin resistant Ehrlich ascites carcinoma. The concentration of daunorubicin required to inhibit 50% of DNA synthesis in daunorubicin-sensitive tumour is approximately one half the concentration required to inhibit an equivalent amount of DNA synthesis in the daunorubicin-resistant variant ($P<0.05$). The addition of CsA to daunorubicin shifts the daunorubicin $IC_{50}$ of thymidine incorporation of EA/DR cells to levels that are characteristic of the drug sensitive tumour. Although the thymidine inhibition assay may not directly reflect cellular viability, we have previously found it to correlate well with *in vivo* response (Slater *et al.*, 1982). *In vivo*, the

**Figure 1** CsA effects on daunorubicin $IC_{50}$ values in daunorubicin sensitive (a) and daunorubicin resistant (b) Ehrlich ascites carcinoma (EA). Cells were washed and resuspended in RPMI 1640 at a concentration of $2.5 \times 10^6$ ml$^{-1}$. Cell aliquots of 1.6 ml were incubated with 0.2 ml daunorubicin HCl (final concentration 0–12 $\mu$g ml$^{-1}$) and 0.2 ml cyclosporin A at a final concentration of 0 (●–●), 3.3 $\mu$g ml$^{-1}$ (■–■), and 13.2 $\mu$g ml$^{-1}$ (×–×) for 1 h at 37°C, washed twice and resuspended in 1.8 ml RPMI. Other details as in **Materials and methods**. Values are plotted as percent inhibition of [$^3$H]-thymidine incorporation compared to control in the absence of daunorubicin.

**Table I** Cyclosporin A reversal of daunorubicin resistance *in vivo*.

| *Tumour line* | *Drug regimen* | *Mean survival* $\pm$ *s.d. (days)* | *Long survivors (60 days)* | *Chi square* | *P* |
|---|---|---|---|---|---|
| (a) EA/DS | — | 18.4±0.6 | 0/5 | | |
| (b) EA/DS | CsA (80 mg kg$^{-1}$) | 24.1±0.7 | 0/10 | | |
| (c) EA/DS | Daunorubicin | >60 | 10/10 | | |
| (d) EA/DS | Daunorubicin | | | | |
| | CsA (80 mg kg$^{-1}$) | >60 | 9[a]/10 | | |
| (e) EA/DR | — | 19.0±1.0 | 0/5 | | |
| (f) EA/DR | CsA (80 mg kg$^{-1}$) | 24.0±2.6 | 0/10 | | |
| (g) EA/DR | Daunorubicin | 21.1±2.6 | 0/10 | 20 *vs* (c) | <0.001 |
| (h) EA/DR | Daunorubicin | | | 20 *vs* (g) | <0.001 |
| | CsA (80 mg kg$^{-1}$) | >60 | 10/10 | 0 *vs* (c), 1.1 *vs* (d) | >0.1 |
| (i) ES/DR | Daunorubicin | | | 20 *vs* (g) | <0.001 |
| | CsA (50 mg kg$^{-1}$) | >60 | 10/10 | 0 *vs* (c), 1.1 *vs* (d) | >0.1 |
| (j) EA/DR | Daunorubicin | | | 20 *vs* (g) | <0.001 |
| | CsA (25 mg kg$^{-1}$) | >60 | 10/10 | 0 *vs* (c), 1.1 *vs* (d) | >0.1 |
| (k) EA/DR | Daunorubicin | | | 8.5 *vs* (g) | <0.01 |
| | CsA (12.5 mg kg$^{-1}$) | 53.9±9.1[b] | 6/10 | 5 *vs* (c), 3 *vs* (d) | <0.02, <0.05 |
| (l) EA/DR | Daunorubicin | | | 5 *vs* (g), 6.4 *vs* (d) | <0.02 |
| | CsA (5 mg kg$^{-1}$) | 51.2±9.8[b] | 4/10 | 8.5 *vs* (c) | <0.01 |

EA/DS and EA/DR signify daunorubicin sensitive and resistant tumour lines, respectively. The treatment regimen consists of daunorubicin 0.3 mg kg$^{-1}$ i.p. daily for five doses, starting 24 h after the inoculation of 0.2 ml i.p. of undiluted malignant ascites harvested from preterminal BALB/c host mice. Total CsA treatment regimens given either alone or simultaneously with daunorubicin in five divided doses are indicated; [a]Indicates the death of one animal in this group at day 5 with diarrhoea. Chi square values compare the frequency of 60 day survival between the indicated groups; [b]Indicates mean survival calculated using 60 day survival for each long survivor.

addition of CsA to daunorubicin in the treatment of EA/DR bearing host mice also restores responses to daunorubicin. When CsA is added to the daunorubicin treatment of the resistant subline, the reduced survival of host mice bearing daunorubicin resistant EA treated with daunorubicin alone returns to the survival characteristic of host mice bearing daunorubicin sensitive EA.

Although the doses of CsA used in these *in vivo* murine studies are similar to those previously employed in humans (Biggs *et al.*, 1983; Kennedy *et al.*, 1985) the use of intraperitoneal CsA in the treatment of an ascitic tumour would favour high local drug concentrations. It is of interest, however, that tissue levels of CsA determined at postmortem examination in patients maintained on CsA for over one week until death range from several hundred to over 7000 ng g$^{-1}$ of tissue wet weight (Ried *et al.*, 1983). These values compare favourably to the 3300 ng ml$^{-1}$ CsA concentration demonstrated to reverse daunorubicin resistance in EA *in vitro*. Although the efficacy of CsA in reversing drug resistance of human tumours *in vivo* has not yet been demonstrated, we do note important *in vitro* activity of CsA in daunorubicin resistant human acute lymphatic leukaemia. CsA completely reverses 50-fold primary resistance to vincristine and 5-fold cross resistance to daunorubicin in a pleiotropic drug resistant subline of GM3639 human T cell acute lymphatic leukaemia – Human Genetic Mutant Cell Repository, Camden, New Jersey (Slater *et al.*, 1986).

The mechanism by which CsA restores responses to daunorubicin and to vincristine is unclear, but may relate to its ability to inhibit calmodulin (Colombani *et al.*, 1985). Verapamil and calmodulin inhibitors have been shown to correct the enhanced active efflux of vinca alkaloids and anthracycline antibiotics characteristic of drug resistant tumour cells resulting in increased cellular drug retention (Tsuruo *et al.*, 1981; 1982). However, Kessel & Corbett (1985) were unable to demonstrate a correlation between adriamycin uptake and adriamycin resistance in murine solid tumours rendered resistant to adriamycin by *in vivo* drug exposure. We were similarly unable to detect significant differences in daunorubicin uptake between our daunorubicin-sensitive and daunorubicin-resistant Ehrlich ascites carcinoma subline and drug sensitive *versus* pleiotropic drug resistant human acute lymphatic leukaemia subline (Slater *et al.*, 1986), suggesting that the mechanism of CsA effect lies beyond the modification of drug transport. Since the acquisition of equimolar concentrations of anthracyclines by anthracycline resistant compared to anthracycline sensitive tumour cells fails to restore equivalent cytotoxic drug effect to these cells (Kessel & Wilberding, 1985*b*; Sikic *et al.*, 1985), the mechanism by which calcium channel blocking agents and calmodulin inhibitors restore drug sensitivity must extend beyond drug retention. It has been shown that adriamycin is cytotoxic when the drug is bound to polygluteraldehyde microspheres or to agarose, which prevents its entry into tumour cells and suggests that cytotoxicity occurs as a cell surface phenomenon (Tritton & Yee, 1982; Rogers *et al.*, 1983). The mechanism of anthracycline antibiotic resistance relates to the cell membrane as well, since recent reports show that pleiotropic drug resistant cells possess characteristic cell membrane glycoprotein alterations in addition to the enhanced active drug efflux described above (Kartner *et al.*, 1983*a,b*).

The studies of LeGrue *et al.* (1983) raise the possibility that CsA might function in the plasma membrane in a manner similar to that of lipid soluble anaesthetics by increasing lipid fluidity and uncoupling electrochemical action potentials. Since drug retention alone cannot account for the effects of calcium channel blocking drugs and calmodulin inhibitors on vinca alkaloid and anthracycline antibiotics in pleiotropic drug resistant cells, it has been suggested that these agents, and it now appears that CsA, may enhance intracellular drug binding or promote favourable chemotherapeutic drug interactions at the membrane level (Beck, 1983; Kessel & Wilberding, 1985*b*).

We wish to thank Dr. Harry Wallerstein for his continuous encouragement and the Marcia Slater Society for Research in Leukemia for financial support.

### References

BECK, W.T. (1983). Vinca alkaloid-resistant phenotype in cultured human leukemic lymphoblasts. *Cancer Treat. Rep.*, **67**, 875.

BIGGS, J., ATKINSON, K., CONCANNON, A. & 10 others (1983). Bone marrow transplantation in 33 patients with malignant blood diseases and severe aplastic anaemia. *Med. J. Aust.*, **2**, 120.

COLOMBANI, P.M., ROBB, A. & HESS, A.D. (1985). Cyclosporin A binding to calmodulin: A possible site of action on T lymphocytes. *Science*, **228**, 337.

KARTNER, N., RIORDAN, J.R. & LING, V. (1983*a*). Cell surface P-glycoprotein associated with multidrug resistance in mammalian cell lines. *Science*, **221**, 1285.

KARTNER, N., SHALES, M., RIORDAN, J.R. & LING, V. (1983*b*). Daunorubicin-resistant Chinese hamster ovary cells expressing multidrug resistance and a cell surface P-glycoprotein. *Cancer Res.*, **43**, 4413.

KENNEDY, M.S., DEEG, H.J., STORB, R. & 10 others (1985). Treatment of acute graft-versus-host disease after allogeneic marrow transplantation. Randomized study comparing corticosteroids and cyclosporine. *Am. J. Med.*, **78**, 978.

KESSEL, D. & CORBETT, T. (1985). Correlations between anthracycline resistance, drug accumulation and membrane glycoprotein patterns in solid tumors of mice. *Cancer Lett.*, **28**, 187.

KESSEL, D. & WILBERDING, C. (1985*a*). Promotion of daunorubicin uptake and toxicity by the calcium antagonist tiapamil and its analogs. *Cancer Treat. Rep.*, **69**, 673.

KESSEL, D. & WILBERDING, C. (1985*b*). Anthracycline resistance in P388 murine leukemia and its circumvention by calcium antagonists. *Cancer Res.*, **45**, 1687.

LEGRUE, S.J., FRIEDMAN, A.W. & KAHAN, B.D. (1983). Binding of cyclosporin by human lymphocytes and phospholipid vesicles. *J. Immunol.*, **131**, 712.

REID, M., GIBBONS, S., KWOK, D., VAN BUREN, C.T., FLECHNER, S. & KAHAN, B.D. (1983). Cyclosporine levels in human tissues of patients treated for one week to one year. *Transplant. Proc.*, **15**, 2434.

ROGERS, K.E., CARR, B.I. & TOKES, Z.A. (1983). Cell surface-mediated cytotoxicity of polymer-bound adriamycin against drug-resistant hepatocytes. *Cancer Res.*, **43**, 2741.

SIKIC, B.I., ETIZ, B.B., HARKER, W.G., ANDERSON, R.L. & HAHN, G.M. (1985). Mechanisms of multidrug (pleiotropic) resistance in the human sarcoma cell line MES-SA. *Proc. Am. Assoc. Cancer Res.*, **26**, 1346a (*Abstract*).

SKOVSGARRD, T., DANO, K. & NISSEN, N.I. (1984). Chemosensitizers counteracting acquired resistance to anthracyclines and vinca alkaloids in vivo. A new treatment principle. *Cancer Treat. Rev.*, **11**, (Suppl A): 63.

SLATER, L.M., MURRAY, S.L. & WETZEL, M.W. (1982). Verapamil restoration of daunorubicin responsiveness in daunorubicin-resistant Ehrlich ascites carcinoma. *J. Clin. Invest.*, **70**, 1131.

SLATER, L.M., SWEET, P., STUPECKY, M. & GUPTA, S. (1986). Cyclosporin A reverses vincristine and daunorubicin resistance in acute lymphatic leukemia in vitro. *J. Clin. Invest.*, (In Press).

TRITTON, T.R. & YEE, G. (1982). The anticancer agent adriamycin can be actively cytotoxic without entering cells. *Science*, **217**, 248.

TSURUO, T., IIDA, H., TSUKAGOSHI, S. & SAKURAI, Y. (1982). Increased accumulation of vincristine and adriamycin in drug-resistant P388 tumor cells following incubation with calcium antagonists and calmodulin inhibitors. *Cancer Res.*, **42**, 4730.

TSURUO, T., IIDA, H., TSUKAGOSHI, S. & SAKURAI, Y. (1981). Overcoming of vincristine resistance in P388 leukemia in vivo and in vitro through enhanced cytotoxicity of vincristine and vinblastine by verapamil. *Cancer Res.*, **41**, 1967.